# In Situ Synthesis of Ru/TiO_2−_ *_x_* @TiCN Ternary Heterojunctions for Enhanced Sonodynamic and Nanocatalytic Cancer Therapy

**DOI:** 10.1002/advs.202307029

**Published:** 2023-11-30

**Authors:** Yin Zhao, Bo Yuan, Lang Yan, Zhiwei Wang, Zheng Xu, Bijiang Geng, Xiang Guo, Xiongsheng Chen

**Affiliations:** ^1^ Spine Center Department of Orthopedics Shanghai Changzheng Hospital Naval Medical University Shanghai 200003 China; ^2^ Department of Health Toxicology Faculty of Naval Medicine Naval Medical University Shanghai 200433 China; ^3^ School of Environmental and Chemical Engineering Shanghai University Shanghai 200444 China

**Keywords:** nanocatalytic therapy, nanozymes, Ru nanoparticles, sonodynamic therapy, TiCN

## Abstract

Albeit nanozymes‐based tumor catalytic therapy (NCT) relies on endogenous chemical reactions that could achieve tumor microenvironment (TME)‐specialized reactive oxygen species (ROS) production, the unsatisfactory catalytic activity of nanozymes accompanied by complex TME poses a barrier to the therapeutic effect of NCT. Herein, a one‐step in situ synthesis strategy is reported to construct ternary Ru/TiO_2−_ *_x_* @TiCN heterojunctions through oxidative conversion of TiCN nanosheets (NSs) to TiO_2−_ *_x_* NSs and reductive deposition of Ru^3+^ to Ru nanoparticles. The narrow bandgap and existence of heterojunctions enhance the ultrasound‐activated ROS generation of Ru/TiO_2−_ *_x_* @TiCN because of the accelerated electron transfer and inhibits electron–hole pair recombination. The augmented ROS production efficiency is achieved by Ru/TiO_2−_ *_x_* @TiCN with triple enzyme‐like activities, which amplifies the ROS levels in a cascade manner through the catalytic decomposition of endogenous H_2_O_2_ to relieve hypoxia and heterojunction‐mediated NCT, as well as depletion of overexpressed glutathione. The satisfactory therapeutic effects of Ru/TiO_2−_ *_x_* @TiCN heterojunctions are achieved through synergetic sonodynamic therapy and NCT, which achieve the complete elimination of tumors without recurrence. This strategy highlights the potential of in situ synthesis of semiconductor heterojunctions as enhanced sonosensitizers and nanozymes for efficient tumor therapy.

## Graphical abstract

A one‐step in situ synthesis strategy is explored to construct ternary Ru/TiO_2−_ *_x_* @TiCN heterojunctions. The narrow bandgap and existence of heterojunctions enhance the ultrasound‐activated reactive oxygen species (ROS) generation of Ru/TiO_2−_ *_x_* @TiCN. Ru/TiO_2−_ *_x_* @TiCN exhibits amplified ROS production through the Ti^3+^‐mediated peroxidase‐like catalytic reaction and the Ti^4+^‐mediated glutathione‐peroxidase‐like catalytic reaction. Complete tumor eradication without recurrence is achieved through synergetic sonodynamic and nanocatalytic therapy.

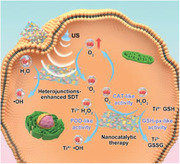

## 1 Introduction

Osteosarcoma is an aggressive malignant tumor that threatens the survival of young adults and their quality of life.^[^ ^1^ ^]^ Current clinical cancer therapy modalities, including surgery, chemotherapy, and radiotherapy, are inadequate to eradicate malignant tumors because of the remarkable systematic toxicity.^[^ ^2^, ^3^, ^4^, ^5^ ^]^ Versatile emerging tumor therapeutic modalities based on exogenous stimulation or endogenous chemical reactions to produce reactive oxygen species (ROS) for killing cancer cells have been widely explored to replace traditional treatments.^[^ ^6^, ^7^, ^8^, ^9^, ^10^ ^]^ Among these modalities, nanozyme‐based tumor catalytic therapy (NCT), which relies on enzymatic reactions in a tumor microenvironment (TME), could combine the advantages of nanomaterials and natural enzymes, catalyzing the generation of highly cytotoxic ROS and achieving a satisfactory therapeutic effect.^[^ ^11^, ^12^, ^13^, ^14^, ^15^, ^16^ ^]^ Unlike low‐efficiency and unstable natural enzymes, several features of low‐cost, high stability, tunable activity, large‐scale production, and high reproducibility enhance the catalytic activity of nanozymes even under harsh environments.^[^ ^17^, ^18^, ^19^ ^]^ Previously reported nanozymes, including noble metals,^[^ ^20^, ^21^, ^22^ ^]^ metal oxide or sulfide,^[^ ^23^, ^24^ ^]^ metal‐organic framework,^[^ ^25^, ^26^, ^27^ ^]^ and carbon‐based nanomaterials,^[^ ^28^, ^29^ ^]^ have been observed to imitate the functions of natural peroxidase (POD), catalase (CAT), and glutathione peroxidase (GSH‐px), exhibiting TME regulation abilities for amplified ROS production. The doubtful biocompatibility of metal‐based nanozymes, however, commonly restrains their tumor therapeutic efficacy. In addition, these reported nanozymes still suffer from low catalytic activity because of their single‐enzyme mimetic activity.^[^ ^20^, ^30^ ^]^ Therefore, it is imperative to develop nanozymes with multiple enzyme‐like catalytic activities, which not only could mimic POD to catalyze H_2_O_2_ to hydroxyl radicals (•OH) but also possess GSH‐px‐like activity to avoid the consumption of generated •OH.

In addition to exploring nanozymes with multiple enzyme‐like catalytic activities, combining NCT with other tumor therapeutic modalities, such as photothermal or photodynamic therapy (PTT or PDT), or sonodynamic therapy (SDT), is a promising strategy to achieve efficient tumor therapy.^[^ ^11^, ^31^, ^32^, ^33^ ^]^ By utilizing near‐infrared (NIR) laser to irradiate photothermal agents or photosensitizers to generate hyperthermia or ROS, PTT or PDT has been regarded as a noninvasive therapeutic modality and was approved by the U.S. Food and Drug Administration (FDA) for clinical trials to treat tumors.^[^ ^34^, ^35^, ^36^, ^37^, ^38^ ^]^ However, the limited penetration depth of the NIR laser even in the second NIR region (≈1–2 cm) makes PTT or PDT suitable only for superficial tumor therapy.^[^ ^39^, ^40^, ^41^ ^]^ By comparison, SDT triggered by ultrasound (US) possesses a larger penetration depth (>10 cm), higher therapeutic efficiency, and lower side effects, which have permitted SDT as an alternative to phototherapy for treating deep‐seated tumors.^[^ ^42^, ^43^, ^44^, ^45^, ^46^ ^]^ Unfortunately, the poor aqueous stability, potential phototoxicity, unstable chemical property of traditional organic sonosensitizers, and wide bandgap, as well as fast electron–hole pair recombination of inorganic nanomaterials, could result in low ROS yield.^[^ ^32^, ^47^, ^48^, ^49^, ^50^, ^51^, ^52^, ^53^, ^54^ ^]^ Hence, seeking a semiconductor sonosensitizer with a narrow bandgap and further constructing the heterostructure to inhibit the electron–hole recombination has the potential to significantly improve the ROS generation efficiency of inorganic sonosensitizers.^[^ ^55^, ^56^, ^57^, ^58^ ^]^ In addition, the ROS generation efficiency induced by oxygen‐dependent SDT is severely limited by hypoxia in the TME.^[^ ^12^, ^59^, ^60^, ^61^, ^62^, ^63^ ^]^ Researchers have reported that intratumoral O_2_ supplements could be improved by nanozymes with CAT‐like catalytic activity.^[^ ^19^, ^64^, ^65^, ^66^, ^67^, ^68^ ^]^ Therefore, endowing sonosensitizers with triple enzyme‐like catalytic activity could achieve cascade amplification of ROS production and break therapeutic resistance by relieving tumor hypoxia and depleting overexpressed GSH.

Recently, transition metal carbonitrides have received significant interest in electrocatalytic and energy storage fields because of their excellent thermal stability and high electronic conductivity.^[^ ^69^, ^70^, ^71^ ^]^ Compared with the widely reported Ti_3_C_2_ MXene, TiCN nanomaterials possess a narrow bandgap because of the presence of vacancies in the lattice structure of TiCN, which has shown promising potential as an enhanced inorganic sonosensitizer. Moreover, TiCN with the presence of mixed valences of Ti^3+^ and Ti^4^ is expected to exhibit multiple enzyme‐like catalytic activities including Ti^3+^‐mediated POD‐like catalytic reaction to generate cytotoxic •OH and the Ti^4+^‐mediated GSH‐px‐like catalytic reaction to eliminate overexpressed GSH, where Ti_3_C_2_ MXene did not contain Ti^3+^. As far as we know, however, TiCN‐based nanomaterials have not been reported to be employed as sonosensitizers or nanozymes for SDT or NCT. Apart from exploring the semiconductor sonosensitizers, constructing heterojunctions based on TiCN nanomaterials could further improve their SDT performances because of the inhibition of electron–hole pair recombination. Recently, many heterostructure sonosensitizers have been fabricated by depositing metal nanoparticles or inorganic nanomaterials onto the surface of other semiconductor sonosensitizers.^[^ ^57^, ^72^, ^73^, ^74^ ^]^ However, these post‐loading strategies for forming heterojunctions often require multistep reactions, which could result in low yields of heterojunctions and a large waste of raw materials. Moreover, the obtained heterojunctions may not have a matched bandgap, leading to low‐efficiency SDT properties.

**Scheme 1:**
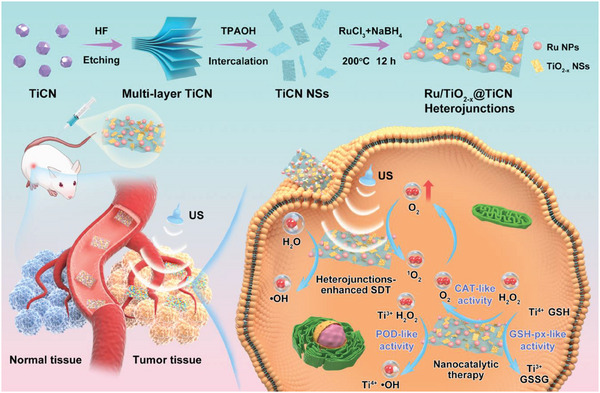
Schematic illustration of the in situ synthesis of Ru/TiO_2−_ *_x_* @TiCN ternary heterojunctions for enhanced SDT and NCT.

In this work, we employed a one‐step in situ synthesis strategy to fabricate ternary Ru/TiO_2−_ *_x_* @TiCN heterojunctions that possess excellent US‐activated ROS generation ability and triple enzyme‐like catalytic activities. Through a facile hydrothermal reaction, the ternary Ru/TiO_2−_ *_x_* @TiCN heterojunctions were formed by the oxidative conversion of TiCN nanosheets (NSs) to TiO_2−_ *_x_* NSs with abundant oxygen vacancies and the reductive deposition of Ru^3+^ to Ru nanoparticles on the surface of TiCN NSs. As a high‐efficiency nanozyme, Ru/TiO_2−_ *_x_* @TiCN heterojunctions were discovered to exhibit amplified ROS production through the Ti^3+^‐mediated POD‐like catalytic activity to generate cytotoxic •OH and the Ti^4+^‐mediated GSH‐px‐like catalytic activity to eliminate overexpressed GSH compared with the pristine TiCN NSs. As an enhanced sonosensitizer, the obtained ternary Ru/TiO_2−_ *_x_* @TiCN displayed improved ROS generation efficiency through the heterojunction‐improved electron–hole separation. In addition, the SDT performance of Ru/TiO_2−_ *_x_* @TiCN was enhanced through its CAT‐like catalytic activity, which rapidly converted intracellular H_2_O_2_ into O_2_ thereby relieving tumor hypoxia. Considering these advantageous features, the ternary Ru/TiO_2−_ *_x_* @TiCN heterojunctions with augmented sonodynamic properties and triple enzyme‐like catalytic activities achieved complete tumor eradication through the synergistic SDT and NCT (**Scheme** **1**).

## 2 Results and Discussion

### 2.1 Preparation and Characterization of Ru/TiO_2−_ *_x_* @TiCN

**Figure 1:**
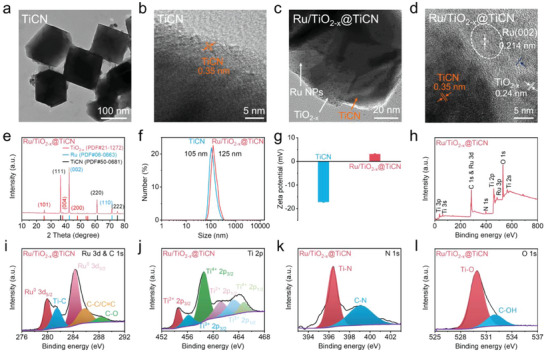
Preparation and characterization of Ru/TiO_2−_ *_x_* @TiCN. a–d) Transmission electron microscopy (TEM) and high‐resolution transmission electron microscopy (HRTEM) images of TiCN and Ru/TiO_2−_ *_x_* @TiCN. e–g) X‐ray powder diffraction (XRD) patterns (e), dynamic light scattering (DLS) (f), and Zeta potential measurements (g) of TiCN and Ru/TiO_2−_ *_x_* @TiCN. h–l) Survey X‐ray photoelectron spectroscopy (XPS) (h), high‐resolution Ru 3d and C 1s (i), Ti 2p (j), N 1s (k), and O 1s (l) spectra of Ru/TiO_2−_ *_x_* @TiCN.

A modified liquid‐phase exfoliation method was utilized to synthesize TiCN NSs through hydrofluoric acid (HF) etching and tetrapropyl ammonium hydroxide (TPAOH) delamination. The transmission electron microscopy (TEM) image presented in **Figure** **1**a indicates that the exfoliated TiCN revealed a lamellar structure with a size of ≈100 nm. The high‐resolution TEM image with higher magnification suggested that TiCN NSs possessed a lattice spacing of 0.35 nm (Figure 1b), which corresponded to the (100) plane of TiCN. Ru/TiO_2−_ *_x_* @TiCN heterojunctions were then synthesized from TiCN NSs through a facile one‐step in situ method in the presence of RuCl_3_ and NaBH_4_. The uniform dispersion of Ru NPs on the surface of TiCN NSs was detected in the TEM image of Ru/TiO_2−_ *_x_* @TiCN heterojunctions (Figure 1c). Apart from the Ru NPs, we also observed the TiO_2−_ *_x_* NSs on the surface of TiCN NSs. Additionally, high‐resolution transmission electron microscopy (HRTEM) images of Ru/TiO_2−_ *_x_* @TiCN heterojunctions revealed three different lattice spacings of 0.35, 0.24, and 0.214 nm (Figure 1d), which could be attributed to TiCN NSs, TiO_2−_ *_x_* NSs, and Ru NPs, respectively. We also performed the TEM element mapping of Ru/TiO_2−_ *_x_* @TiCN heterojunctions. Apart from the C, N, and Ti elements attributed to TiCN NSs, the presence of Ru and O elements ascribed to Ru NPs and TiO_2−_ *_x_* NSs were also detected in the element mapping of Ru/TiO_2−_ *_x_* @TiCN heterojunctions (Figure S1, Supporting Information), suggesting the successful formation of Ru NPs and TiO_2−_ *_x_* NSs on the surface of TiCN NSs. We also captured atomic‐force microscopy (AFM) images of TiCN NSs and Ru/TiO_2−_ *_x_* @TiCN heterojunctions. As depicted in Figure S2a,b, Supporting Information, the height of TiCN NSs was determined to be ≈2 nm, which indicated the sheet structure of TiCN NSs. For Ru/TiO_2−_ *_x_* @TiCN heterojunctions, the height was measured to be ≈7–10 nm (Figure S2c,d, Supporting Information), which suggested the successful formation of Ru NPs and TiO_2−_ *_x_* NSs on the surface of TiCN NSs. The size of the prepared TiCN NSs and Ru/TiO_2−_ *_x_* @TiCN heterojunctions was further determined by dynamic light scattering (DLS). The hydrodynamic diameter of Ru/TiO_2−_ *_x_* @TiCN was measured to be 125 nm (Figure 1f), which was slightly higher than that of the pristine TiCN NSs (105 nm) because of the formation of heterojunctions. We also investigated the change of surface charge before and after the formation of Ru/TiO_2−_ *_x_* @TiCN heterojunctions. Figure 1g exhibits that the zeta potential of TiCN was reversed from negative (−17.1 ± 0.3 mV) to positive charge (3 ± 0.3 mV). The stability of Ru/TiO_2−_ *_x_* @TiCN heterojunctions in different mediums was then evaluated. Figure S3, Supporting Information, exhibits the clear solution of Ru/TiO_2−_ *_x_* @TiCN dispersed in deionized (DI) water, fetal bovine serum (FBS), or Dulbecco's modified eagle medium (DMEM) after 7 days, indicating the excellent stability of Ru/TiO_2−_ *_x_* @TiCN in different medium after long‐term storage. The stability of Ru/TiO_2−_ *_x_* @TiCN dispersed in different mediums was also assessed by determining their hydrodynamic diameter after long‐term storage. As depicted in Figure S4, Supporting Information, no significant changes in the hydrodynamic diameter of Ru/TiO_2−_ *_x_* @TiCN dispersed in DI water could be observed after 7 days.

We then performed X‐ray powder diffraction (XRD), Fourier transform infrared spectroscopy (FTIR), and X‐ray photoelectron spectroscopy (XPS) characterizations to investigate the chemical structure and element composition of Ru/TiO_2−_ *_x_* @TiCN heterojunctions. The XRD patterns of pristine TiCN NSs are shown in Figure S5, Supporting Information, which revealed five diffraction peaks corresponding to the (311), (220), (222), (200), and (111) planes of TiCN NSs (PDF#50‐0681). Moreover, the five diffraction peaks of TiCN became sharper after etching, indicating the high purity of the obtained TiCN NSs. For Ru/TiO_2−_ *_x_* @TiCN heterojunctions, apart from the diffraction peak of TiCN, we also found that the (002) and (110) planes corresponded to Ru NPs (PDF#06‐0663) and the (101), (004), and (200) planes corresponded to TiO_2−_ *_x_* NSs (PDF#21‐1272) (Figure 1e), forcefully verifying the successful formation of Ru NPs and TiO_2−_ *_x_* NSs on the surface of TiCN NSs. Additionally, the presence of TiO_2−_ *_x_* NSs on TiCN was demonstrated by the FTIR spectra (Figure S6, Supporting Information), which suggested that Ti─O and C═O were formed in Ru/TiO_2−_ *_x_* @TiCN heterojunctions. The Ru 3d peak was detected in Ru/TiO_2−_ *_x_* @TiCN for their survey XPS spectrum in addition to the Ti 2p, C 1s, N 1s, and O 1s peaks attributed to TiCN NSs (Figure 1h and Figure S7, Supporting Information), verifying that the Ru NPs were formed on the surface of TiCN NSs. The formation of Ru NPs on TiCN NSs was further confirmed by their high‐resolution Ru 3d spectrum (Figure 1i), which revealed two peaks centered at 279.8 and 284.3 eV, which corresponded to Ru^0^ 3d_5/2_ and Ru^0^ 3d_3/2_, respectively, indicating the formation of Ru NPs on the surface of TiCN NSs. The presence of TiO_2−_ *_x_* in heterojunctions was verified by the high‐resolution Ti 2p and O 1s spectra (Figure 1j,l), which illustrated the presence of Ti^3+^ and Ti─O, respectively. In addition to Ti^3+^, the existence of Ti^2+^ and Ti^4+^ were also detected in Ru/TiO_2−_ *_x_* @TiCN, which was similar to that of pristine TiCN (Figure S7, Supporting Information). For the high‐resolution N 1s spectrum of heterojunctions and TiCN NSs, two peaks at 396.4 and 399.2 eV were detected, which could be attributed to Ti─N and C─N, respectively. These above characterization results clearly demonstrated that the Ru NPs and TiO_2−_ *_x_* NSs were formed on the surface of TiCN NSs.

### 2.2 Enhanced Sonodynamic Properties of Ru/TiO_2−_ *_x_* @TiCN

**Figure 2:**
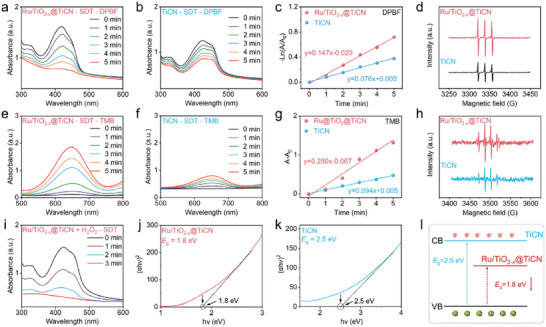
Enhanced sonodynamic properties of Ru/TiO_2−_ *_x_* @TiCN. a,b,e,f) ^1^O_2_ and •OH generated by Ru/TiO_2−_ *_x_* @TiCN (a,e) and TiCN (b,f) upon the treatment of US irradiation. c,g) Calculating the rate constant of ^1^O_2_ and •OH production in the presence of Ru/TiO_2−_ *_x_* @TiCN or TiCN. d,h) Electron spin resonance (ESR) spectra of the ^1^O_2_ (d) and •OH (h) generated by Ru/TiO_2−_ *_x_* @TiCN and TiCN. i) Time‐dependent ^1^O_2_ generation evaluation of Ru/TiO_2−_ *_x_* @TiCN in the presence of H_2_O_2_. j,k) Tauc plot of Ru/TiO_2−_ *_x_* @TiCN (j) and TiO_2_ (k). l) Schematic illustration of the decreased bandgap of Ru/TiO_2−_ *_x_* @TiCN compared with TiO_2_.

After confirming the fabrication of Ru/TiO_2−_ *_x_* @TiCN, we then performed sonodynamic activity measurements to compare the ROS generation capabilities before and after the construction of heterojunctions. **Figure** **2**a,b reveals that the decreased degree in the characteristic absorption peak of DPBF of heterojunctions treated by US was higher than that of TiCN, elucidating that more ^1^O_2_ was produced by Ru/TiO_2−_ *_x_* @TiCN. Moreover, the rate constant of ^1^O_2_ produced by Ru/TiO_2−_ *_x_* @TiCN upon the irradiation of US was 0.147 min^−1^ (Figure 2c), which was 1.9 times higher than that in single‐component TiCN NSs (0.076 min^−1^), which illuminated that the heterojunctions possessed improved sonodynamic properties. Apart from US‐activated ^1^O_2_ generation, the generation efficiency of •OH through Ru/TiO_2−_ *_x_* @TiCN or TiCN was also evaluated using tetramethylbenzidine (TMB) as the colorimetric indicator. Compared with the pristine TiCN NSs, the peak regarding the oxidized derivative of TMB at 652 nm exhibited a larger increase (Figure 2e,f). The enhanced rate constant of •OH production in the heterojunction group triggered by the US was measured to be 0.290 min^−1^ (Figure 2g), which was 3.1 times in comparison with that in the pristine TiCN group (0.094 min^−1^), which clearly demonstrated that the ROS generation efficiency of TiCN nanosheets could be augmented by the formation of heterojunctions. The enhanced ROS generation of heterojunctions compared with the pristine NSs was then investigated by electron spin resonance (ESR) spectroscopy using 2,2,6,6‐tetramethylpiperidine (TEMP) and 5, 5‐dietyl‐1‐pyrroline‐*N*‐oxide (DMPO) as the ^1^O_2_ and •OH trapping agent, respectively. As illustrated in Figure 2d,h, a higher ^1^O_2_ and •OH signal could be detected in the Ru/TiO_2−_ *_x_* @TiCN group in comparison with the TiCN group, which provided more evidence for the improved ^1^O_2_ and •OH production efficiency for heterojunctions.

We then investigated the energy band structure of Ru/TiO_2−_ *_x_* @TiCN heterojunctions by measuring their bandgap to elucidate the mechanism of enhanced ROS generation. Figure S8, Supporting Information, shows the enhanced absorption spectrum of Ru/TiO_2−_ *_x_* @TiCN, which ranged from visible to the NIR region compared with the pristine TiCN NSs can be observed in Figure S8, Supporting Information. The bandgap (*E*_g_) of Ru/TiO_2−_ *_x_* @TiCN and TiCN was then calculated to be 1.8 and 2.5 eV, respectively. The decreased *E*_g_ of heterojunctions compared with pristine NSs indicated that the electron in the valence band of Ru/TiO_2−_ *_x_* @TiCN was activated more easily, and more electrons could react with O_2_ to produce the ^1^O_2_. Moreover, more holes left in the conduction band of Ru/TiO_2−_ *_x_* @TiCN could react with H_2_O to generate •OH, thereby achieving enhanced ROS generation ability. In addition to the decreased bandgap, the presence of heterojunctions within Ru/TiO_2−_ *_x_* @TiCN could further improve their ROS generation efficiency because the US‐activated electric could transfer from TiCN to TiO_2−_ *_x_* or Ru, thereby inhibiting the recombination of electron–hole pairs.

### 2.3 Enhanced Triple Enzyme‐Like Catalytic Activities of Ru/TiO_2−_ *_x_* @TiCN

**Figure 3:**
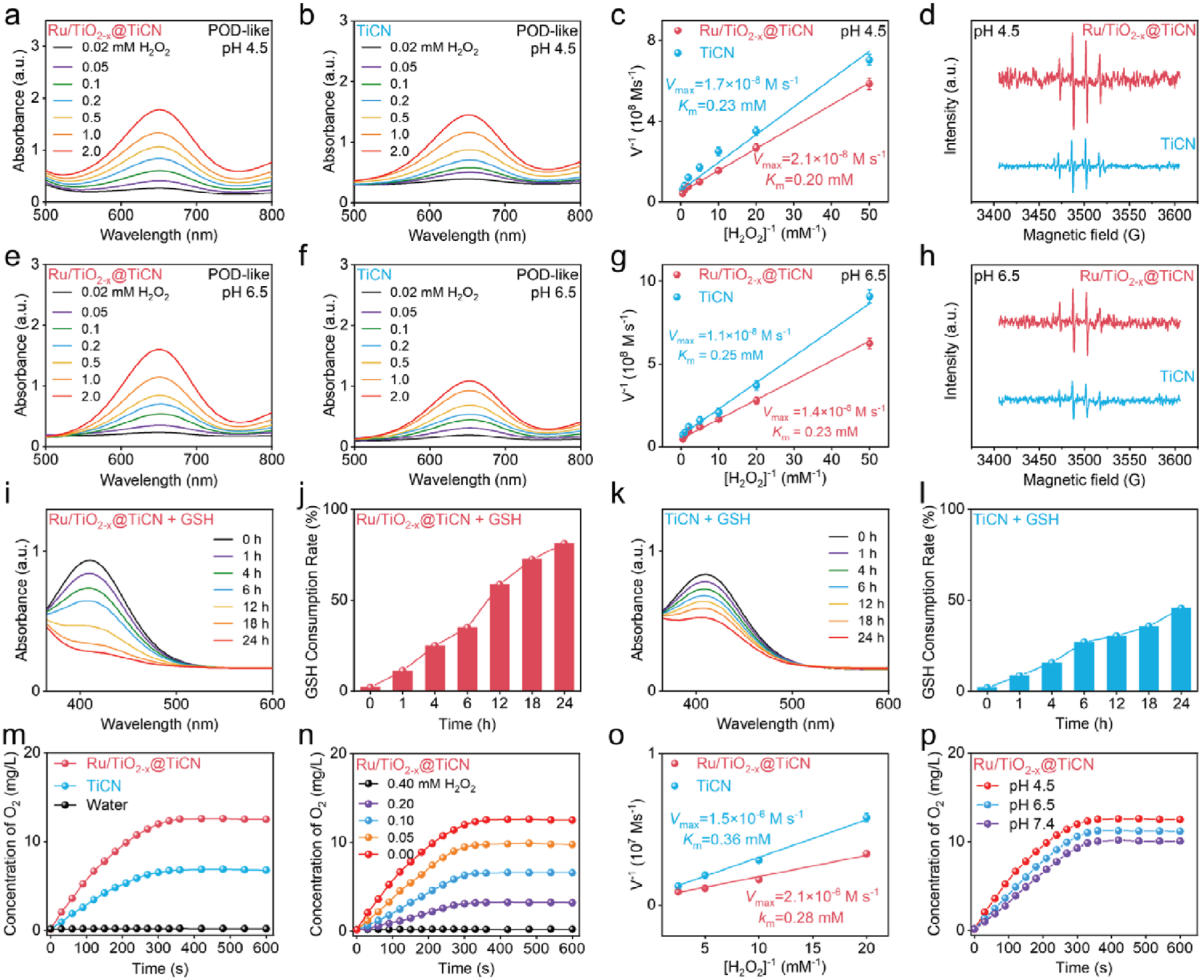
Enhanced triple enzyme‐like catalytic activities of Ru/TiO_2−_ *_x_* @TiCN. a,b,e,f) Absorption change of TMB solution incubated with Ru/TiO_2−_ *_x_* @TiCN (a,e) and TiCN (b,f) in the presence of H_2_O_2_ with varied concentrations. c,g) Comparison of POD‐like catalytic activity of Ru/TiO_2−x_@TiCN and TiCN at different pH. d,h) ESR spectra of the •OH generated by Ru/TiO_2−_ *_x_* @TiCN (d) and TiCN (h) in the presence of H_2_O_2_ (2 mm). i–l) GSH‐px‐like catalytic activity evaluation of Ru/TiO_2−_ *_x_* @TiCN (i,j) and TiCN (k,l). m) CAT‐like catalytic activity evaluation of Ru/TiO_2−_ *_x_* @TiCN and TiCN. n,p) CAT‐like catalytic activity evaluation of Ru/TiO_2−_ *_x_* @TiCN in the presence of H_2_O_2_ with varied concentrations (0–0.4 mm) or at different pH. o) Comparison of POD‐like catalytic activity of Ru/TiO_2−_ *_x_* @TiCN and TiCN.

Given the existence of Ru NPs and the mixed valence state of Ti^3+^ and Ti^4+^ in heterojunctions, we then investigated the triple enzyme‐like catalytic activities of Ru/TiO_2−_ *_x_* @TiCN. As the H_2_O_2_ concentration increased, the absorbance of TMB was observed to rise in the presence of Ru/TiO_2−_ *_x_* @TiCN (**Figure** **3**a), which manifested their efficient POD‐like catalytic activity at a mildly acidic pH of 4.5. Compared with the TiCN group, the TMB absorption in the Ru/TiO_2−_ *_x_* @TiCN group was much more pronounced (Figure 3b), which suggested that the formation of heterojunctions would enhance the POD‐like catalytic activity of pristine NSs. The Michaelis–Menten kinetics analysis was performed to further quantify the enhanced catalytic activity of heterojunctions by comparing the maximal reaction velocity (*V*_max_) and Michaelis–Menten constant (*K*_M_). Figure 3c reveals that the *V*_max_ and *K*_M_ of Ru/TiO_2−_ *_x_* @TiCN were determined to be 2.1 × 10^−8^ m s^−1^ and 0.20 mm, respectively, which were higher than those of pristine TiCN NSs (*V*_max_ = 1.7 × 10^−8^ m s^−1^ and *K*_M_ = 0.23 mm). In addition to the colorimetric method, we also utilized ESR spectra to verify the enhanced POD‐like catalytic activity of Ru/TiO_2−_ *_x_* @TiCN. As depicted in Figure 3d, the stronger •OH signal could be detected in the Ru/TiO_2−_ *_x_* @TiCN compared with TiCN, suggesting that the formation of Ru NPs and TiO_2−_ *_x_* NSs with abundant oxygen vacancies could significantly enhance the catalytic activity of TiCN NSs. Considering the influence of pH on the catalytic performance, we then conducted POD‐like catalytic activity evaluation at pH 6.5 and 7.4. For pH 6.5, similar results of heterojunction‐enhanced POD‐like catalytic activity can be detected, which revealed the higher absorbance of oxidic TMB in the Ru/TiO_2−_ *_x_* @TiCN group compared with the pristine TiCN group (Figure 3e,f). Moreover, the higher *V*_max_ and lower *K*_M_ were calculated in the heterojunction group (Figure 3g), suggesting that the POD‐like catalytic activity of TiCN NSs could be amplified by the formation of Ru NPs and TiO_2−_ *_x_* NSs on the surface of TiCN NSs. ESR spectra further exhibited that a higher •OH signal could be detected in Ru/TiO_2−_ *_x_* @TiCN at pH 6.5 compared with TiCN (Figure 3h). Furthermore, the catalytic activity of Ru/TiO_2−_ *_x_* @TiCN at pH 6.5 was weaker than that at pH 4.5, illustrating that the POD‐like catalytic reaction occurred easily in acidic conditions. We also investigated the influence of Ru NPs on the catalytic activity of Ru/TiO_2−_ *_x_* @TiCN. Unlike the preparation of Ru/TiO_2−_ *_x_* @TiCN, TiO_2−_ *_x_* @TiCN was formed by the oxidative conversion of TiCN to TiO_2−_ *_x_* through a facile hydrothermal reaction without the addition of RuCl_3_ and NaBF_4_. Figure S9a,c, Supporting Information, reveals that the absorbance of TMB was observed to rise in the presence of TiO_2−_ *_x_* @TiCN at pH 4.5 or 6.5, suggesting their efficient POD‐like catalytic activity. Compared with pristine TiCN NSs, the lower *K*_M_ was observed in the TiO_2−_ *_x_* @TiCN (Figure S9b,d, Supporting Information), indicating that the formation of heterojunctions within TiO_2−_ *_x_* and TiCN would also improve the POD‐like catalytic activity of TiCN. However, the *K*_M_ of TiO_2−_ *_x_* @TiCN was higher than that of Ru/TiO_2−_ *_x_* @TiCN, demonstrating that the formation of Ru NPs on the surface of TiCN would further enhance the POD‐like catalytic activity of TiCN. Importantly, we did not detect any significant •OH generation in Ru/TiO_2−_ *_x_* @TiCN or TiCN solution in the presence of H_2_O_2_ at pH 7.4 (Figure S10, Supporting Information), indicating that Ru/TiO_2−_ *_x_* @TiCN or TiCN could not exhibit POD‐like catalytic activity under normal physiological conditions. These results forcefully confirmed that the catalytic activity of single TiCN NSs could be improved by the doping of metal nanoparticles or metal ions with mixed valence state.

The GSH‐px‐like catalytic activities of Ru/TiO_2−_ *_x_* @TiCN or TiCN were also evaluated using 5,5′‐dithiobis‐(2‐nitrobenzoic acid) (DTNB) as the colorimetric indicator. The GSH‐px‐like catalytic activities were investigated by mixing Ru/TiO_2−_ *_x_* @TiCN or TiCN with GSH followed by measuring the absorption change of the mixture solution. The effectiveness of TiCN in depleting GSH was demonstrated by a decrease in DTNB absorbance at 412 nm as the incubation time increased (Figure 3k,l), which could be attributed to the Ti^4+^‐mediated GSH‐px‐like activity of TiCN NSs. Moreover, a greater decline in the DTNB peak in Ru/TiO_2−_ *_x_* @TiCN solution could be detected in comparison with single‐component TiCN (Figure 3i,j and Figure S11, Supporting Information), which confirmed the amplifying GSH‐px‐like activity of TiCN NSs after forming Ru/TiO_2−_ *_x_* @TiCN heterojunctions.

We also evaluated the CAT‐like catalytic activity of Ru/TiO_2−_ *_x_* @TiCN compared with pristine TiCN. We utilized the dissolved oxygen meter to measure the O_2_ concentration in the Ru/TiO_2−_ *_x_* @TiCN or TiCN solution with the addition of the H_2_O_2_ solution. Figure 3m exhibits that the O_2_ generation efficiency of Ru/TiO_2−_ *_x_* @TiCN was much higher than that of pristine TiCN at the same H_2_O_2_ concentration (0.4 mm), demonstrating the higher CAT‐like catalytic activity of Ru NPs and TiO_2−_ *_x_* NSs co‐loaded TiCN NSs. Moreover, more O_2_ molecules were produced in the higher H_2_O_2_ concentration for Ru/TiO_2−_ *_x_* @TiCN or TiCN (Figure 3n and Figure S12, Supporting Information), indicating their H_2_O_2_ concentration‐dependent O_2_ generation behaviors. Furthermore, the higher *V*_max_ and lower *K*_M_ could be detected in the Ru/TiO_2−_ *_x_* @TiCN group (2.4 × 10^−6^ m s^−1^ and 0.35 mm) compared with that in the pristine TiCN group (1.6 × 10^−6^ m s^−1^ and 0.41 mm) (Figure 3o), forcefully confirming the efficient CAT‐like catalytic activity of heterojunctions. By changing the pH of the reaction system, we also evaluated the pH‐dependent O_2_ generation behaviors of Ru/TiO_2−_ *_x_* @TiCN and TiCN. Figure 3p and Figure S13, Supporting Information, exhibit that more O_2_ molecules were generated by Ru/TiO_2−_ *_x_* @TiCN and TiCN in the acidic condition. These results clearly confirmed that the formation of Ru NPs and TiO_2−_ *_x_* NSs with abundant oxygen vacancies could effectively improve the CAT‐like catalytic activity of pristine TiCN NSs.

Considering that O_2_ molecules played an important role in US‐activated ^1^O_2_ generation, we also investigated whether the ROS generation ability of Ru/TiO_2−_ *_x_* @TiCN could be improved by the augmented O_2_ molecule concentration because of the CAT‐like activity of heterojunctions. Figure 2i reveals that more ^1^O_2_ was produced by Ru/TiO_2−_ *_x_* @TiCN in the presence of H_2_O_2_ under US treatments, demonstrating that the SDT properties of Ru/TiO_2−_ *_x_* @TiCN could be further augmented by the Ru/TiO_2−_ *_x_* @TiCN‐mediated CAT‐like catalytic reaction. The quantitative results presented in Figure S14, Supporting Information, further reveal the higher ^1^O_2_ rate constant in the Ru/TiO_2−_ *_x_* @TiCN + H_2_O_2_ group (0.384 min^−1^) compared with the single Ru/TiO_2−_ *_x_* @TiCN group (0.147 min^−1^).

### 2.4 In Vitro Enhanced SDT and NCT via Ru/TiO_2−_ *_x_* @TiCN

**Figure 4:**
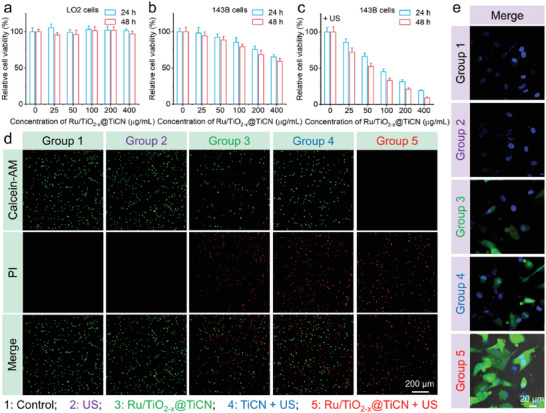
In vitro enhanced SDT and NCT via Ru/TiO_2−_ *_x_* @TiCN. a,b) Cytotoxicity evaluation of Ru/TiO_2−_ *_x_* @TiCN against LO2 (a) or 143B cells (*n* = 6). c) Cytotoxicity evaluation of Ru/TiO_2−_ *_x_* @TiCN against 143B cells under US irradiation. d,e) Live/dead cell (d) and ROS staining (e) evaluation of different treatments against 143B cells.

Encouraged by the amplified sonodynamic and catalytic activities of Ru/TiO_2−_ *_x_* @TiCN, the in vitro heterojunction‐enhanced SDT and NCT through Ru/TiO_2−x_@TiCN were then investigated. The cell uptake behaviors of Ru/TiO_2−_ *_x_* @TiCN labeled by the NIR fluorescent dye indocyanine green (ICG) were initially evaluated by the confocal microscope. The bright red fluorescent signal can be observed in the cytoplasm of 143B or LO2 cells after incubating with Ru/TiO_2−_ *_x_* @TiCN (Figure S15, Supporting Information), illustrating the efficient cellular internalization of heterojunctions. The good biocompatibility of Ru/TiO_2−_ *_x_* @TiCN and TiCN was initially confirmed by the MTT assay (**Figure** **4**a and Figure S16, Supporting Information), suggesting their insignificant cytotoxicity against human normal liver LO2 cells. In contrast, a certain cytotoxic effect of Ru/TiO_2−_ *_x_* @TiCN on human osteosarcoma 143B cells, as shown in Figure 4b, which we attributed to the production of •OH in the tumor cells with higher H_2_O_2_ concentration in comparison with normal cells. Moreover, the relative cell viability of Ru/TiO_2−_ *_x_* @TiCN‐treated 143B cells was lower than that of pristine TiCN (Figure S17, Supporting Information). For combined SDT and NCT, the cytotoxicity of Ru/TiO_2−_ *_x_* @TiCN against 143B cells was further increased (Figure 4c), which demonstrated their efficient therapeutic effect. Noted that the therapeutic effect of synergistic SDT and NCT through Ru/TiO_2−_ *_x_* @TiCN was superior to that of TiCN (Figure S18, Supporting Information), which indicated the improved sonodynamic performance and POD‐like catalytic activity of TiCN NSs after forming Ru/TiO_2−_ *_x_* @TiCN heterojunctions.

In addition to the MTT assay, the enhanced SDT and NCT through Ru/TiO_2−_ *_x_* @TiCN were also confirmed by the live/dead cell staining. For US treatment alone, no significant cytotoxic effect can be induced (Figure 4d), indicating the safety of US irradiation at conditions of 50 kHz and 1 W cm^−2^. A slight red fluorescence represented dead cells was detected in the Ru/TiO_2−_ *_x_* @TiCN alone group, suggesting the therapeutic effect of POD‐like catalytic reaction induced by heterojunctions. Notably, the strongest red and no green fluorescence signals were detected in the Ru/TiO_2−_ *_x_* @TiCN + US group, demonstrating that the collaborative SDT and NCT induced by heterojunctions possessed a higher cytotoxic effect compared with a single NCT.

We performed ROS staining of 143B cells after different treatments to evaluate intracellular ROS levels. Figure 4e exhibits that no significant green fluorescence that represented ROS was detected in 143B cells treated with PBS and US alone, confirming that a single US treatment would not generate ROS to kill cells. For comparison, a slight green fluorescence could be observed in the Ru/TiO_2−_ *_x_* @TiCN alone group and the TiCN + US group, suggesting that the single Ru/TiO_2−_ *_x_* @TiCN‐mediated NCT and TiCN‐mediated SDT/NCT could generate a certain amount of ROS to kill cancer cells. We also performed the ROS staining of LO2 cells after treatment with Ru/TiO_2−_ *_x_* @TiCN. As depicted in Figure S19, Supporting Information, no significant green fluorescence that represented ROS was detected in the LO2 cells treated with PBS, US alone, and Ru/TiO_2−_ *_x_* @TiCN alone, demonstrating that the single Ru/TiO_2−_ *_x_* @TiCN‐mediated NCT could not generate ROS because of the low H_2_O_2_ concentration in the normal cells. Significantly, the strongest green fluorescence signal in 143B cells was observed in the Ru/TiO_2−_ *_x_* @TiCN + US group, demonstrating that Ru/TiO_2−_ *_x_* @TiCN in the presence of US irradiation produced most of the ROS compared with the other groups. We also performed the flow cytometry apoptosis assay according to the typical Annexin V‐FITC and PI staining protocol. Figure S20, Supporting Information, reveals that most cells with apoptosis and necrosis were detected in the Ru/TiO_2−_ *_x_* @TiCN + US group compared with the other groups, which suggested that Ru/TiO_2−_ *_x_* @TiCN‐mediated SDT and NCT could generate ROS and then induce cell apoptosis and necrosis. These results demonstrated that Ru/TiO_2−_ *_x_* @TiCN could integrate several therapeutic modalities of SDT and NCT onto one platform to achieve satisfying anticancer efficiency.

### 2.5 In Vivo Enhanced SDT and NCT via Ru/TiO_2−_ *_x_* @TiCN

**Figure 5:**
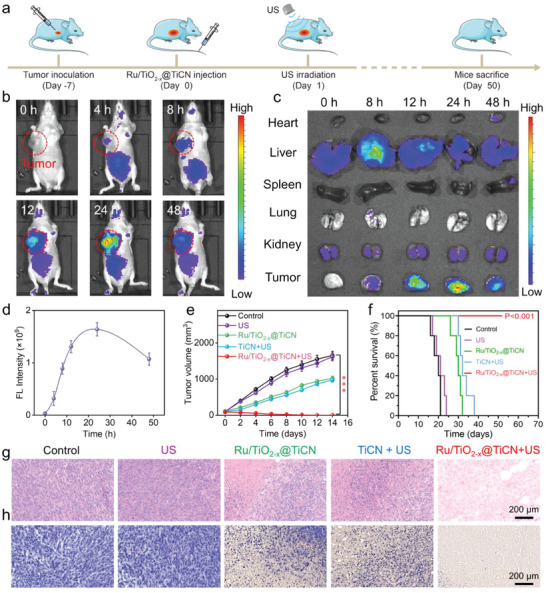
In vivo enhanced SDT and NCT through Ru/TiO_2−_ *_x_* @TiCN. a) Schematic illustration of the in vivo combined SDT and NCT through Ru/TiO_2−_ *_x_* @TiCN. b–d) NIR imaging and corresponding quantitative results of 143B tumor‐bearing mice at the in vivo and ex vivo levels after intravenous injection of Ru/TiO_2−_ *_x_* @TiCN. e–h) Tumor volume (e), survival (f), H&E staining (g), and TUNEL staining (h) of mice after different treatments including control, US, Ru/TiO_2−_ *_x_* @TiCN, TiCN + US, and Ru/TiO_2−_ *_x_* @TiCN + US. (*n* = 5 and ***p < 0.001 (ANOVA)).

Motivated by the admirable in vitro therapeutic effect of Ru/TiO_2−_ *_x_* @TiCN through collaborative SDT and NCT, we then performed animal experiments to investigate their synergetic antitumor efficiency in vivo. The biodistribution of Ru/TiO_2−_ *_x_* @TiCN was initially evaluated using NIR fluorescence imaging following intravenous injection of ICG labeled Ru/TiO_2−_ *_x_* @TiCN to mice. The gradually increased fluorescence signal at the tumor sites after injection of ICG@Ru/TiO_2−_ *_x_* @TiCN is shown in **Figure** **5**b,d, indicating that Ru/TiO_2−_ *_x_* @TiCN could accumulate in tumors. Meanwhile, the ex vivo NIR imaging results further indicated that the strongest signal of Ru/TiO_2−_ *_x_* @TiCN accumulated in tumor tissues was observed at 24 h post‐injection (Figure 5c and Figure S21, Supporting Information). We then utilized inductively coupled plasma mass spectrometry (ICP‐MS) to investigate the distribution of Ru/TiO_2−_ *_x_* @TiCN in major organs and tumors after intravenous injection at different times. As presented in Figure S22, Supporting Information, Ti content in the tumor tissues was higher than the liver tissues at 24 h post‐injection, confirming the obvious enrichment of Ru/TiO_2−_ *_x_* @TiCN in tumors despite partial accumulation in endothelial reticular systems was also detected.

The subcutaneous tumor model was then constructed to investigate the therapeutic effects of combined SDT and NCT through Ru/TiO_2−_ *_x_* @TiCN. After intravenous injection of Ru/TiO_2−_ *_x_* @TiCN for 24 h (Figure 5a), the 143B tumor‐bearing mice were irradiated with US at the condition of 50 kHz and 1 W cm^−2^. The tumor volume of mice in the control and US irradiation alone groups rapidly increased (Figure 5e), illustrating that US irradiation did not induce a significant antitumor effect. In contrast, a slight inhibition effect of tumor growth was detected in the Ru/TiO_2−_ *_x_* @TiCN alone group, attributed to the single therapeutic effect of NCT based on Ru/TiO_2−_ *_x_* @TiCN‐mediated POD‐like catalytic reaction. Notably, complete eradication of tumor tissues was achieved in the Ru/TiO_2−_ *_x_* @TiCN + US group, whereas the inhibition rate of tumor growth in the TiCN + US group was only 41%, demonstrating the formation of Ru NPs and TiO_2−_ *_x_* NSs on the surface of TiCN NSs improved the SDT effect and POD‐like catalytic activity of TiCN NSs. Moreover, the mice in Ru/TiO_2−_ *_x_* @TiCN + US group were all alive after 50 days (Figure 5f), whereas the mice in the control group were alive only for up to 20 days, further confirming the enhanced antitumor effect of Ru/TiO_2−_ *_x_* @TiCN through combined SDT and NCT. The histological analysis of tumor tissues was then conducted to further verify the therapeutic effect of Ru/TiO_2−_ *_x_* @TiCN‐induced combinatorial NCT/SDT using H&E and TUNEL staining. The H&E staining images presented in Figure 5g indicate that the tumors in the Ru/TiO_2−_ *_x_* @TiCN + US group were apoptosis or necrosis. A similar phenomenon was observed in the TUNEL staining images of tumors, suggesting that severe apoptosis/necrosis could be induced by Ru/TiO_2−_ *_x_* @TiCN in the presence of US irradiation (Figure 5h). We then performed the ROS staining of tumors in mice after different treatments to clarify the mechanism of enhanced SDT and NCT using dihydroethidium (DHE) as the ROS probe. Figure S23, Supporting Information, reveals that a slight red fluorescence was detected in the Ru/TiO_2−_ *_x_* @TiCN alone group, which was attributed to the single therapeutic effect of NCT based on the Ru/TiO_2−_ *_x_* @TiCN‐mediated POD‐like catalytic reaction. Significantly, the higher ROS signals were observed in Ru/TiO_2−_ *_x_* @TiCN + US group compared with TiCN + US group, suggesting that the formation of Ru NPs and TiO_2−_ *_x_* NSs on the surface of TiCN NSs improved the SDT effect and POD‐like catalytic activity of TiCN NSs.

We then performed the biodistribution analysis of Ru/TiO_2−_ *_x_* @TiCN at different time points to investigate their metabolic pathway. As shown in Figure S24, Supporting Information, Ru/TiO_2−_ *_x_* @TiCN primarily accumulated in the liver because of the capture of the reticuloendothelial system. At 14 days after injection, no obvious Ti signal could be detected in these organs, indicating that Ru/TiO_2−_ *_x_* @TiCN was eliminated from the mice through the liver. Then, the in vivo biocompatibility of Ru/TiO_2−_ *_x_* @TiCN‐induced synergistic NCT/SDT was assessed by measuring the change in mice weight, detecting hematological tests, and performing histological analysis. Figure S25, Supporting Information, indicates that Ru/TiO_2−_ *_x_* @TiCN under US irradiation would not induce a fluctuation of body weight of mice during the treatment period, demonstrating the insignificant long‐term toxicity of Ru/TiO_2−_ *_x_* @TiCN‐mediated combinatorial SDT and NCT. Moreover, H&E staining of the major organs of mice after different treatments exhibited no significant inflammation or pathological on day 14 (Figure S26, Supporting Information), confirming the insignificant side effects of Ru/TiO_2−_ *_x_* @TiCN. In addition, the hematological tests revealed that the main markers of hepatic and renal function of mice after different treatments were within the normal ranges (Figure S27, Supporting Information), suggesting insignificant inflammation or disease occurrence during the treatment periods. These above results forcefully verified that systemically administrating Ru/TiO_2−_ *_x_* @TiCN under US irradiation possessed good biosafety in vivo.

## 3 Conclusions

In summary, we synthesized ternary Ru/TiO_2−_ *_x_* @TiCN heterojunctions through a one‐step in situ hydrothermal method involving the oxidative conversion of TiCN NSs to TiO_2−_ *_x_* NSs and reductive deposition of Ru^3+^ to Ru nanoparticles. The formation of heterojunctions inhibited electron–hole recombination within Ru/TiO_2−_ *_x_* @TiCN under US irradiation for enhanced sonodynamic activity, showing a higher rate constant of ^1^O_2_ and •OH generation (0.147 and 0. 290 min^−1^), which was 1.9 and 3.1 times that of pristine TiCN NSs (0.076 and 0.094 min^−1^), respectively. The obtained Ru/TiO_2−_ *_x_* @TiCN nanozymes mimicked POD, CAT, and GSH‐px to catalyze the decomposition of endogenous H_2_O_2_ into cytotoxic •OH for boosting oxidative stress and O_2_ generation for relieving hypoxia, as well as depleting of overexpressed GSH. Encouraged by the favorable features of Ru/TiO_2−_ *_x_* @TiCN heterojunctions, a satisfactory therapeutic effect with complete tumor eradication was achieved by the cooperative SDT and NCT.

## 4 Experimental Section

### One‐Step Synthesis of Ru/TiO_2−_ *_x_* @TiCN

The bulk TiCN ceramic (1 g) was mixed with 40 mL of HF (40%) and the mixture was stirred at 25 °C for 3 days. Then, the mixture was added to TPAOH solution (200 mL) and stirred for 2 days to obtain TiCN nanosheets. Subsequently, 200 mg of TiCN nanosheets were added to the 40 mL DI water in the presence of 3 mg mL^−1^ RuCl_3_. 100 mg of NaBF_4_ was added to the mixture, which was then exposed to ultrasonic treatment for 30 min. The mixture was then heated at 200 °C for 12 h in an oven. Ru/TiO_2−_ *_x_* @TiCN was finally obtained by collecting the precipitates after washing the solid sample with DI water and ethanol several times.

### Characterization

TEM and HRTEM images of Ru/TiO_2−_ *_x_* @TiCN and TiCN were obtained from the JEM‐2100F microscope. Rigaku 18 KW D/max‐2550, Nicolet AVATAR 370 FT‐IR, and Kratos Axis Ultra DLD were utilized to carry out the XRD, FTIR, and XPS measurements of Ru/TiO_2−_ *_x_* @TiCN and TiCN, respectively. Agilent Cary 5000 was utilized to detect the absorption spectra of Ru/TiO_2−_ *_x_* @TiCN and TiCN, respectively. Bruker EMXplus was utilized to acquire the ESR spectra of Ru/TiO_2−_ *_x_* @TiCN and TiCN.

### Sonodynamic Performance Evaluation

DPBF (20 µg) or TMB (1 µm) was mixed with Ru/TiO_2−_ *_x_* @TiCN and TiCN solution at the same concentration (0.4 mg mL^−1^) and the US irradiation was performed at the condition of 50 kHz and 1 W cm^−2^. The absorption spectra of the mixture in each group were measured every minute during the US irradiation periods to determine the ^1^O_2_ and •OH generation ability. ESR spectra were also used to measure the ROS production efficiency of Ru/TiO_2−_ *_x_* @TiCN and TiCN using TEMP or DMPO as the trapping agent. 20 µL of TEMP or DMPO were added to Ru/TiO_2−_ *_x_* @TiCN or TiCN and the US treatments were performed for 2 min. The mixture was then exposed to an ESR spectrometer to measure the ^1^O_2_ and •OH generation efficiency of Ru/TiO_2−_ *_x_* @TiCN or TiCN.

### Triple Enzyme‐Like Catalytic Activity Evaluation

The absorption change of TMB or DTNB in each group was measured to determine the POD‐like or GSH‐px‐like catalytic activity of Ru/TiO_2−_ *_x_* @TiCN or TiCN. For POD‐like catalytic activity, 20 µL of TMB was mixed with Ru/TiO_2−_ *_x_* @TiCN or TiCN at the same concentration (400 µg mL^−1^) in the presence of H_2_O_2_ with various concentrations (0–2 mm). The absorption spectra of the mixture were then measured after incubation for 30 min for the determination of POD‐like catalytic activity. Lineweaver–Burk Plot was further utilized to calculate the *K*_M_ and *V*_max_ in each group using Michaelis–Menten fitting. For GSH‐px‐like catalytic activity, GSH (1 mm) was added to Ru/TiO_2−_ *_x_* @TiCN or TiCN solution, and the mixture was incubated for 0–24 h. At different times, 50 µL of the mixture was added to the DTNB solution, and the absorption spectra of the mixture were measured. For CAT‐like catalytic activity, a portable dissolved oxygen meter (JPBJ‐607) was used to detect the O_2_ molecule concentrations in the mixture of Ru/TiO_2−_ *_x_* @TiCN or TiCN in the presence of H_2_O_2_ (0, 50, 100, 200, and 400 µm). Similar to the POD‐like catalytic activity evaluation, the CAT‐like catalytic activity of Ru/TiO_2−_ *_x_* @TiCN or TiCN was analyzed using the Michaelis–Menten fitting.

### Cellular Experiments

LO2 and 143B cells were purchased from Cell Bank, Chinese Academy of Sciences. For the biocompatibility evaluation, Ru/TiO_2−_ *_x_* @TiCN or TiCN with various concentrations (0–400 µg mL^−1^) was incubated with LO2 cells for 24 or 48 h. Based on the provided protocols, a standard MTT assay was performed to evaluate the biocompatibility. For in vitro NCT, a standard MTT assay was performed using 143B cells after treatment with Ru/TiO_2−_ *_x_* @TiCN or TiCN at various concentrations (0–400 µg mL^−1^). For in vitro combined SDT and NCT, Ru/TiO_2−_ *_x_* @TiCN or TiCN was incubated with 143B cells for 4 h and the US irradiation was performed for 5 min. After irradiation, 143B cells were then incubated for another 24 or 48 h. Finally, a standard MTT assay was performed to assess the in vitro SDT and NCT based on the provided protocols.

For live/dead staining and ROS staining, 143B cells were initially seeded in a 6‐well plate and then treated with PBS, US (50 kHz, 1 W cm^−2^), Ru/TiO_2−_ *_x_* @TiCN, TiCN + US, or Ru/TiO_2−_ *_x_* @TiCN + US, respectively. The fluorescence microscope was used to capture the live/dead staining images of 143B cells after staining with calcein AM and PI. The confocal microscope was used to obtain the ROS staining images of 143B cells after staining with DAPI and DCFH‐DA.

### Tumor Model

The nude mice at 5 weeks old were purchased from SLAC (Shanghai, China) and performed the animal experiments based on the permission of the Institutional Animal Care and Use Committee of Shanghai University (SYXK 2019‐0020). 3 × 10^6^ 143B cells were injected into the backside of the mice to establish the subcutaneous tumor model.

### In Vivo NIR Fluorescence Imaging

Ru/TiO_2−_ *_x_* @TiCN were labeled by an NIR dye ICG to achieve the NIR imaging capability. After intravenous injection of ICG@Ru/TiO_2−_ *_x_* @TiCN, the 143B tumor‐bearing mice were imaged by a VIS Lumina III Imaging System (PerkinElmer, USA) to determine the biodistribution of Ru/TiO_2−_ *_x_* @TiCN. The major organs and tumor tissues were harvested at different times and then imaged.

### In Vivo Combined SDT and NCT

After tumor volume reached about 100 mm^3^, the 143B tumor‐bearing mice were assigned to five groups including control, US (50 kHz, 1 W cm^−2^), Ru/TiO_2−_ *_x_* @TiCN, TiCN + US, and Ru/TiO_2−_ *_x_* @TiCN + US. The US irradiation was performed at 24 h post‐injection of Ru/TiO_2−_ *_x_* @TiCN. H&E, TUNEL, and ROS staining of tumor tissues were carried out after US treatment for 24 h. After treatment for 14 days, major organs and tumors were harvested for histopathological analysis and hematological tests. During the treatment periods, the length and width of tumor tissues were measured to calculate the tumor volume using a vernier caliper.

### Statistical Analysis

To ensure the accuracy of the experiments, at least three replicates were performed. All data are presented as mean values ± SD. ANOVA was used for data with multiple comparison groups. All statistical analyses were performed by Origin 2018 and GraphPad Prism 8.0. Statistical significance between the two groups was calculated with a two‐tailed Student's *t*‐test. * denotes a statistical significance (*p < 0.05, **p < 0.01, and ***p < 0.001) between the experimental data of two groups.

## Conflict of Interest

The authors declare no conflict of interest.

## Supporting information

Supporting Information

## Data Availability

The data that support the findings of this study are available from the corresponding author upon reasonable request.
