# Supplementary material for: In Situ Synthesis of Ru/TiO2− x @TiCN Ternary Heterojunctions for Enhanced Sonodynamic and Nanocatalytic Cancer Therapy
Source: Adv Sci (Weinh). 2023 Nov 30;11(4):2307029. doi: 10.1002/advs.202307029 (PMC10811504; doi:10.1002/advs.202307029)
Supplement: Supplementary file 1 — Supporting Information [file ADVS-11-2307029-s001.pdf]

ADVANCED SCIENCE
Open Access

Supporting Information

for *Adv. Sci.*, DOI 10.1002/advs.202307029

# In Situ Synthesis of Ru/$TiO_{2-x}$@TiCN Ternary Heterojunctions for Enhanced Sonodynamic and Nanocatalytic Cancer Therapy

*Yin Zhao, Bo Yuan, Lang Yan, Zhiwei Wang, Zheng Xu, Bijiang Geng*, Xiang Guo* and Xiongsheng Chen**

# In-Situ Synthesis of Ru/$TiO_{2-x}$@TiCN Ternary Heterojunctions for Enhanced Sonodynamic and Nanocatalytic Cancer Therapy

*Yin Zhao, Bo Yuan, Lang Yan, Zhiwei Wang, Zheng Xu, Bijiang Geng*, Xiang Guo*, Xiongsheng Chen**

Y. Zhao, B. Yuan, Z. Wang, Z. Xu, X. Guo, X. Chen

Spine Center, Department of Orthopedics, Shanghai Changzheng Hospital, Naval Medical University, Shanghai, 200003, China

E-mail: gx9601074@smmu.edu.cn (X. Guo), cxspine@smmu.edu.cn (X. Chen)

L. Yan

Department of Health Toxicology, Faculty of Naval Medicine, Naval Medical University, Shanghai, 200433, China

B. Geng

School of Environmental and Chemical Engineering, Shanghai University, Shanghai 200444, China

E-mail: bjgeng1992@shu.edu.cn (B. Geng)

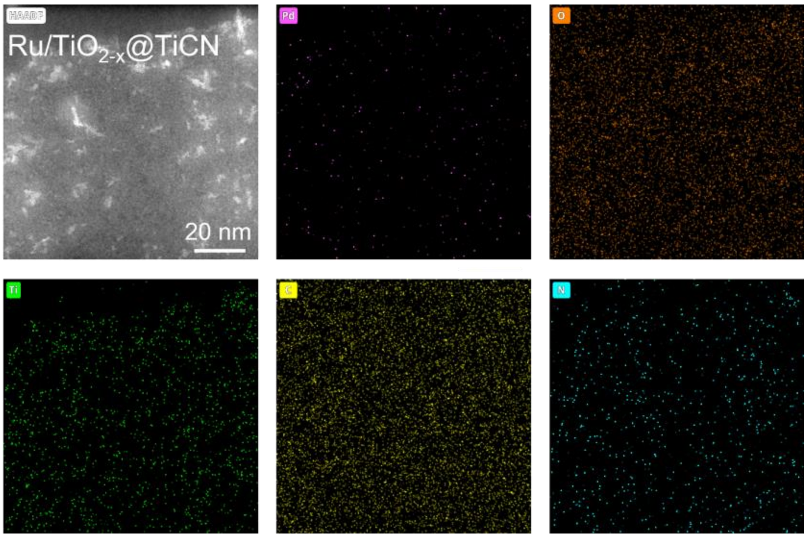


**Figure S1.** TEM element mapping images of Ru/$TiO_{2-x}$@TiCN.

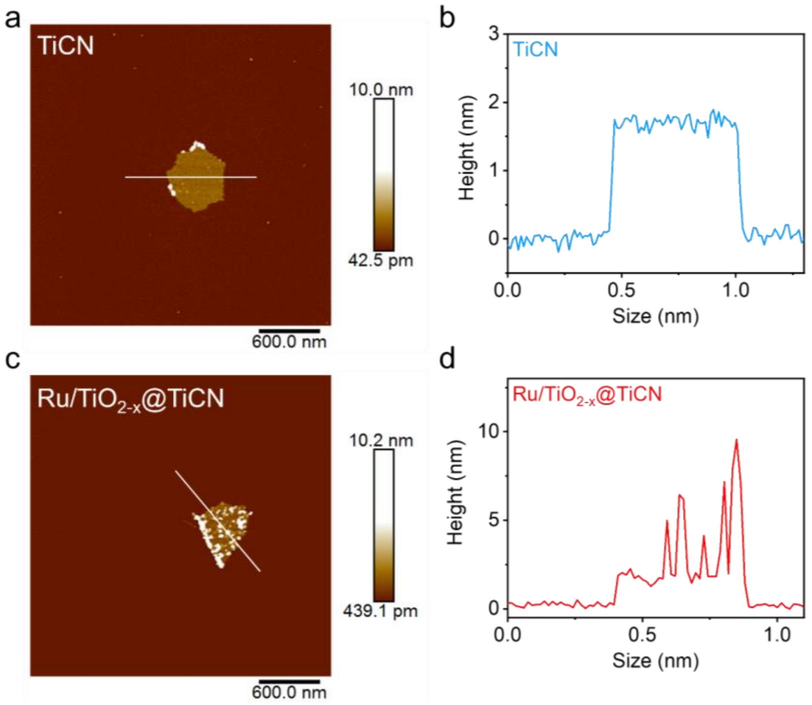


**Figure S2.** AFM image and displaying height profiles of TiCN (a, b) and Ru/$TiO_{2-x}$@TiCN (c, d).

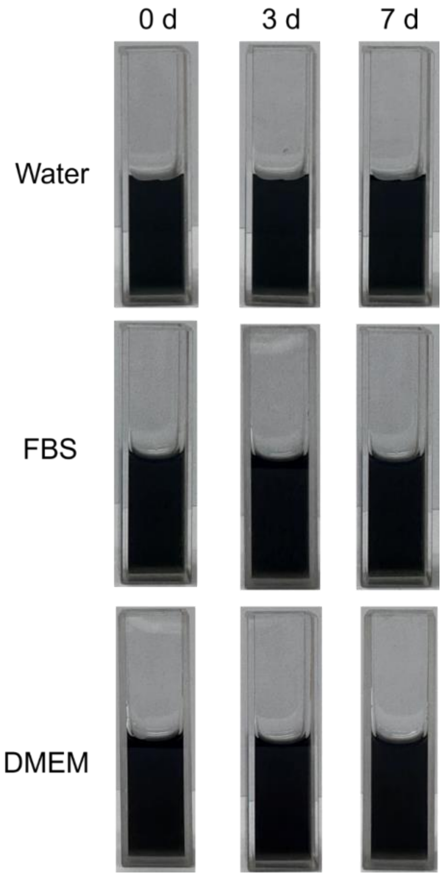


**Figure S3.** Photographs of Ru/$TiO_{2-x}$@TiCN aqueous solution, FBS solution, and DMEM solution stored for different periods of time (0, 3, and 7 days).

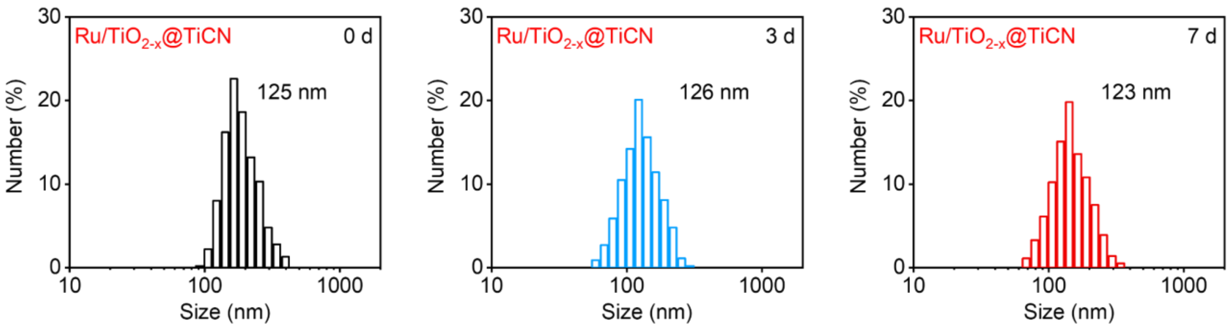


**Figure S4.** The hydrodynamic diameters of Ru/$TiO_{2-x}$@TiCN aqueous solution stored for 0, 3, and 7 days.

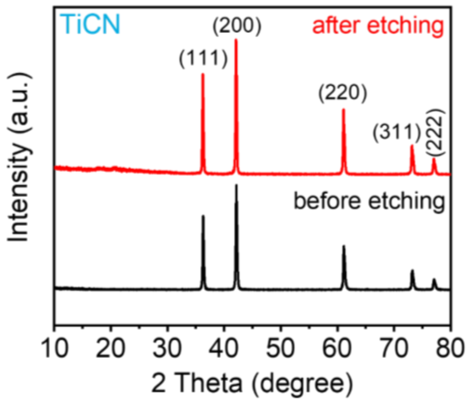


**Figure S5.** XRD patterns of TiCN before and after HF etching.

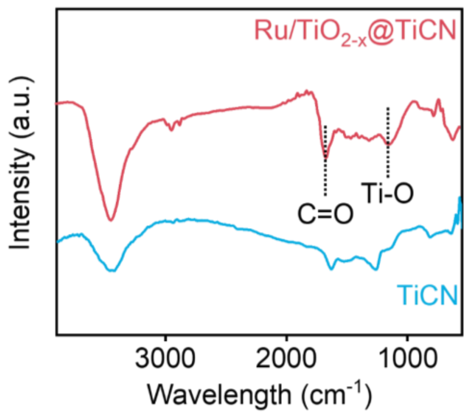


**Figure S6.** FTIR spectra of Ru/$TiO_{2-x}$@TiCN and TiCN.

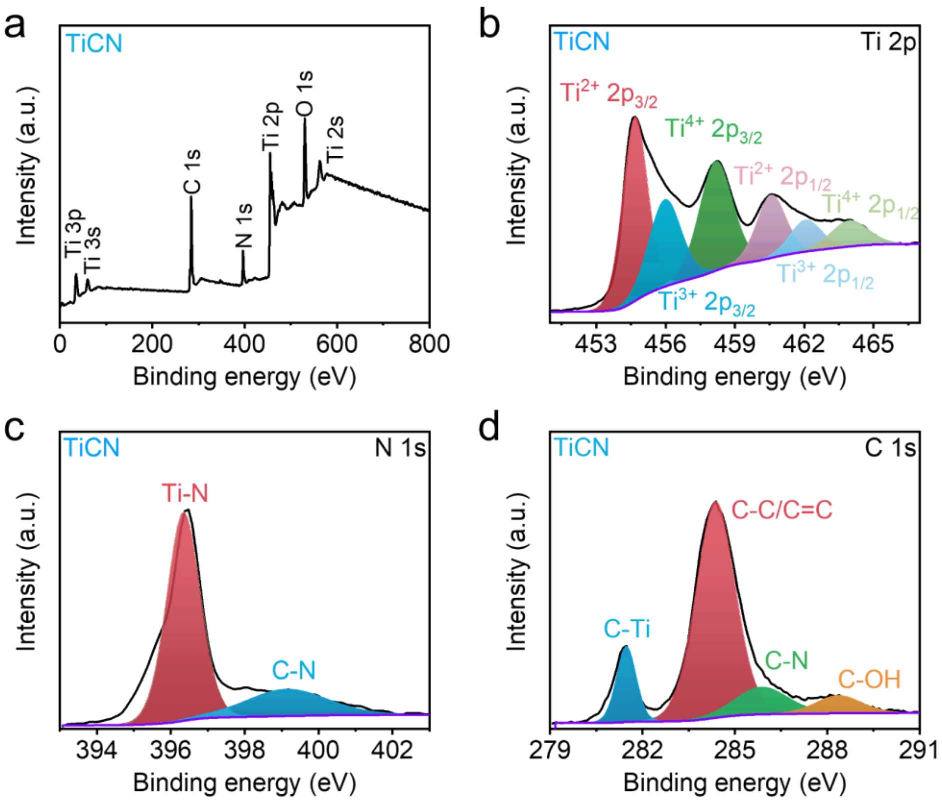


**Figure S7.** (a-d) The survey XPS (a), high-resolution Ti 2p (b), N 1s (c), and C 1s spectra (d) of TiCN NSs.

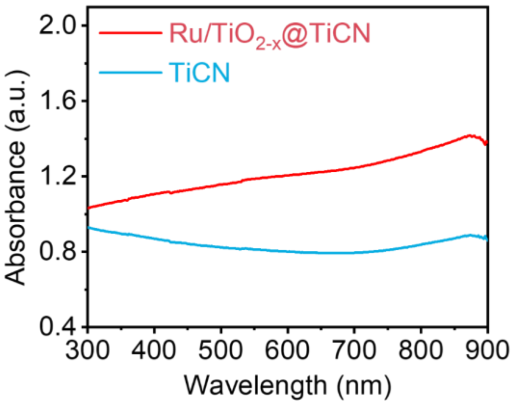


**Figure S8.** Absorption spectra of $Ru/TiO_{2-x}$@TiCN and TiCN.

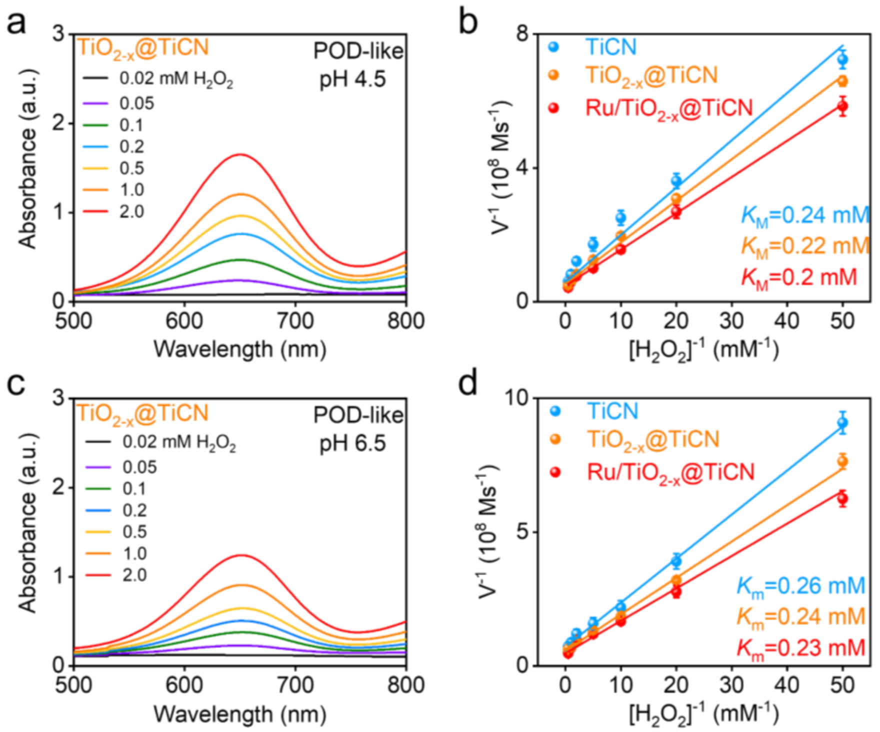


**Figure S9.** (a, c) Absorption change of TMB solution incubated with $TiO_{2-x}$@TiCN in the presence of $H_2O_2$ with varied concentration at pH 4.5 (a) and 6.5 (c). (b, d) Comparison of POD-like catalytic activity of Ru/$TiO_{2-x}$@TiCN, $TiO_{2-x}$@TiCN, and TiCN at pH 4.5 (b) and 6.5 (d).

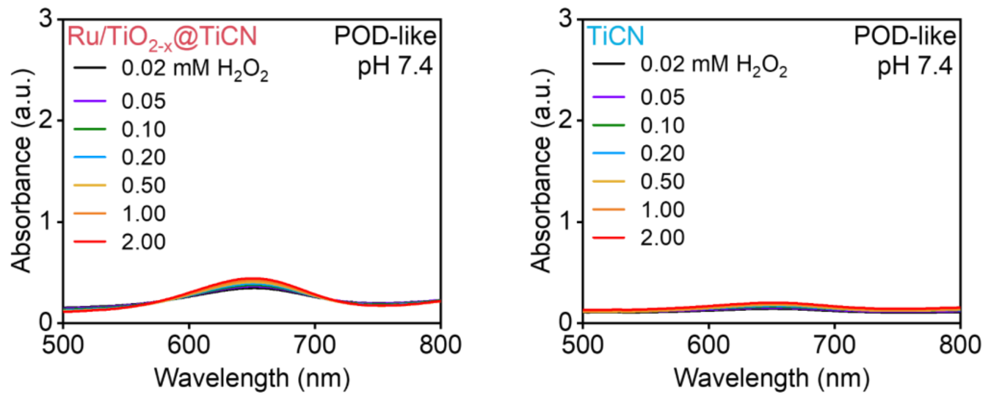


**Figure S10.** Absorption spectra of the oxidized TMB catalyzed by Ru/$TiO_{2-x}$@TiCN or TiCN at pH 7.4.

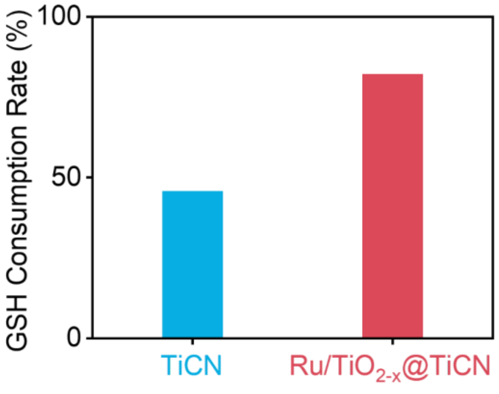


**Figure S11.** The GSH depletion activity evaluation of Ru/$TiO_{2-x}$@TiCN and TiCN.

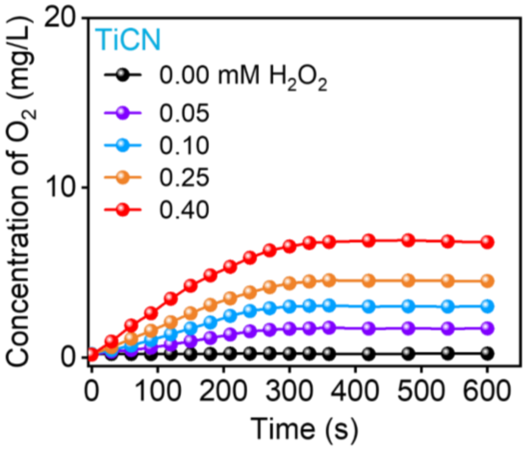


**Figure S12.** $O_2$ generation upon the addition of TiCN (0.4 mg/mL) in the presence of $H_2O_2$ with varied concentrations at pH 4.5.

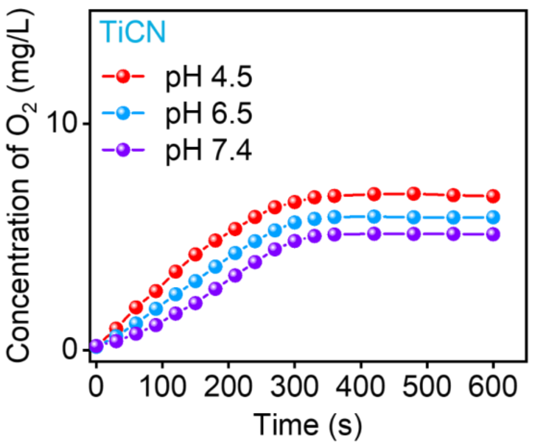


**Figure S13.** $O_2$ generation upon the addition of TiCN (0.4 mg/mL) and $H_2O_2$ (0.40 mM) at varied pH (4.5, 6.5, or 7.4).

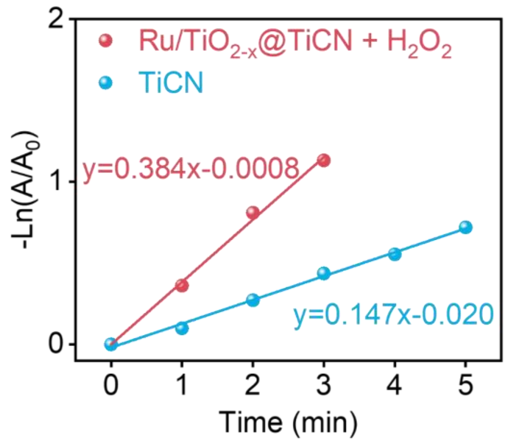


**Figure S14.** The rate constant of $^{1}O_2$ generation of Ru/$TiO_{2-x}$@TiCN and TiCN under US irradiation in the presence of $H_2O_2$ (0.40 mM).

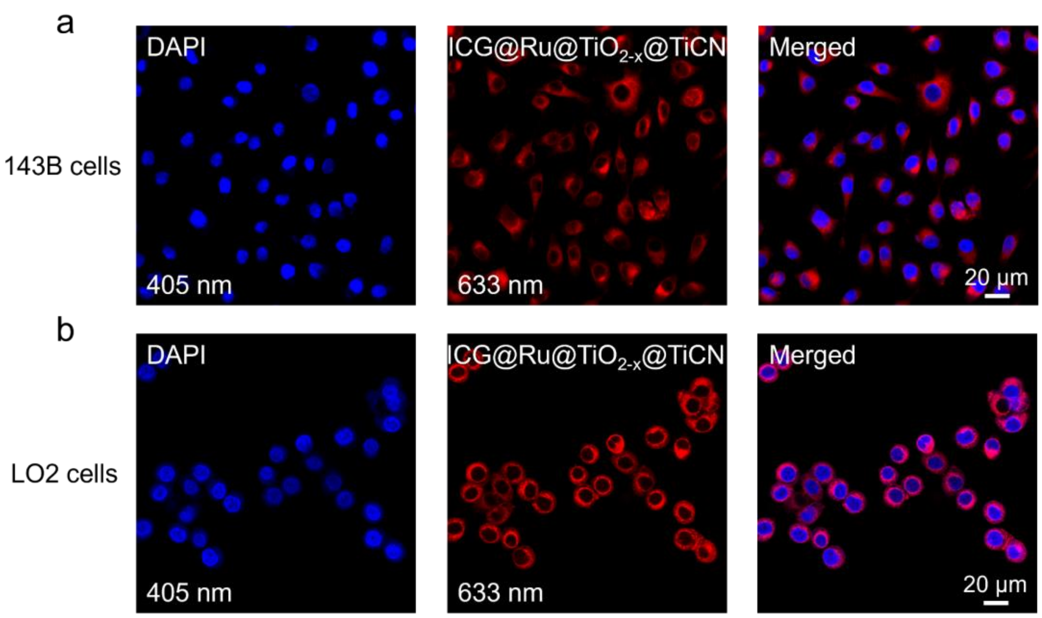


**Figure S15.** DAPI and ICG staining of 143B (a) or LO2 cells (b) incubated with ICG-labeled Ru/$TiO_{2-x}$@TiCN.

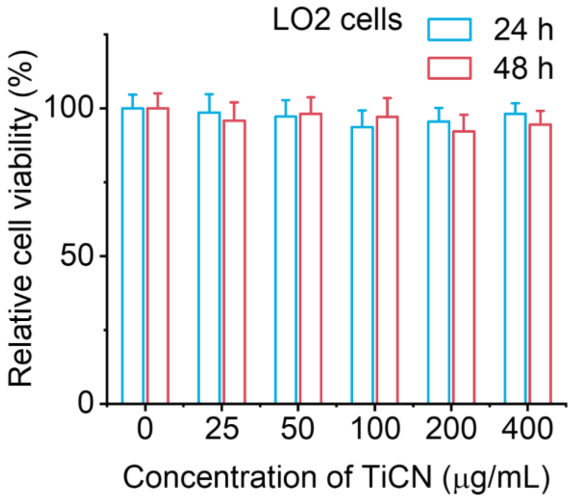


**Figure S16.** Relative cell viabilities of LO2 cells incubated with TiCN with varied concentrations for 24 or 48 h (n=6).

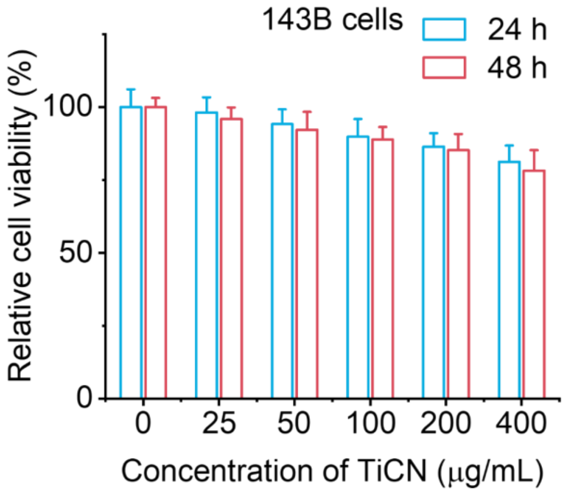


**Figure S17.** Relative cell viabilities of 143B cells incubated with TiCN with varied concentrations for 24 or 48 h (n=6).

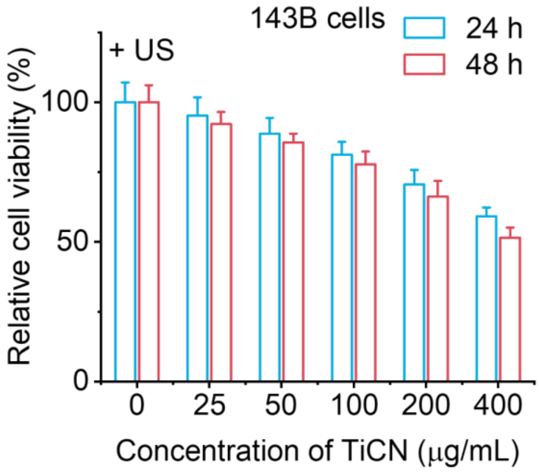


**Figure S18.** Relative cell viabilities of 143B cells incubated with TiCN with varied concentrations for 24 or 48 h in the presence of US irradiation (n=6).

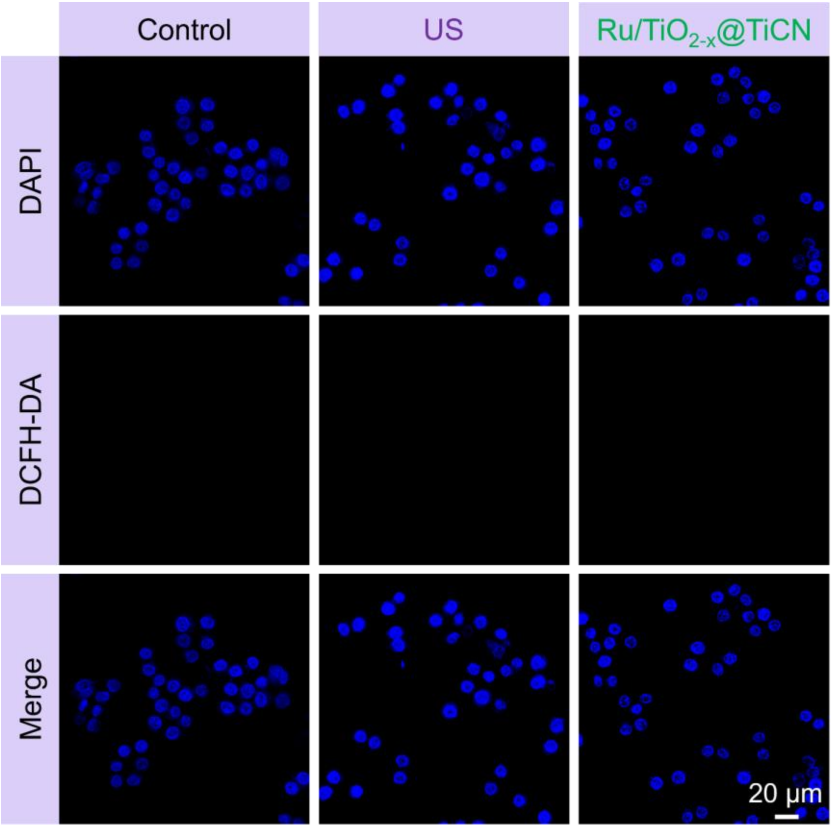


**Figure S19.** ROS staining of LO2 cells after different treatments.

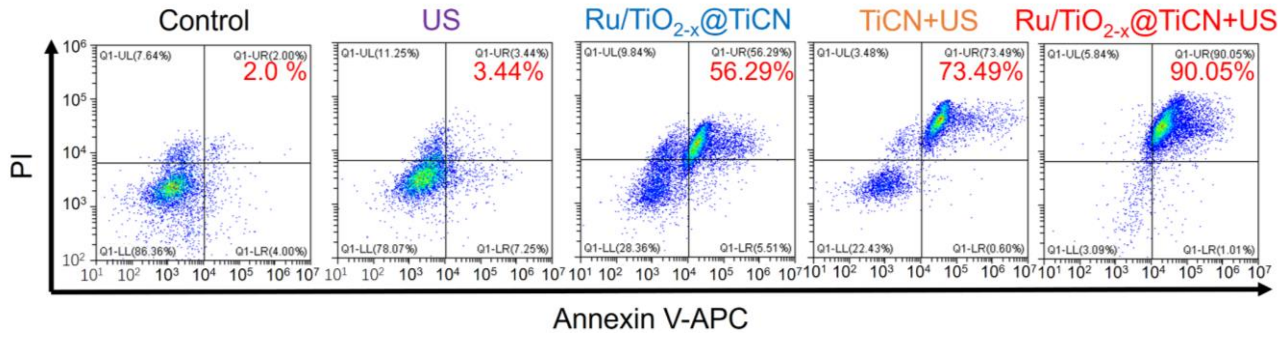


**Figure S20.** Flow cytometry apoptosis assay of 143B cells after different treatments.

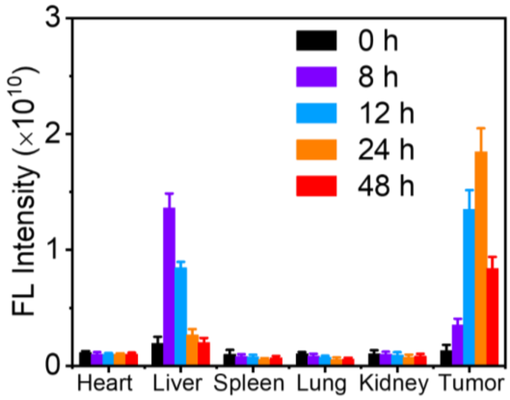


**Figure S21.** Time-dependent NIR fluorescence intensity of ICG-labeled Ru/$TiO_{2-x}$@TiCN in the major organs and tumor tissues based on the fluorescence imaging results (n=5).

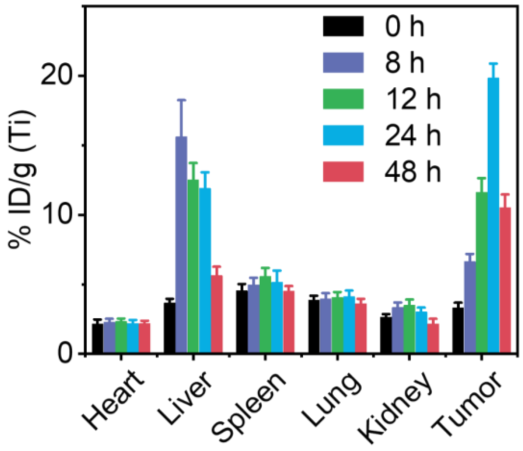


**Figure S22.** Biodistribution measurements of Ru/$TiO_{2-x}$@TiCN in major organs and tumors after intravenous injection of different times determined by ICP-MS (n=5).

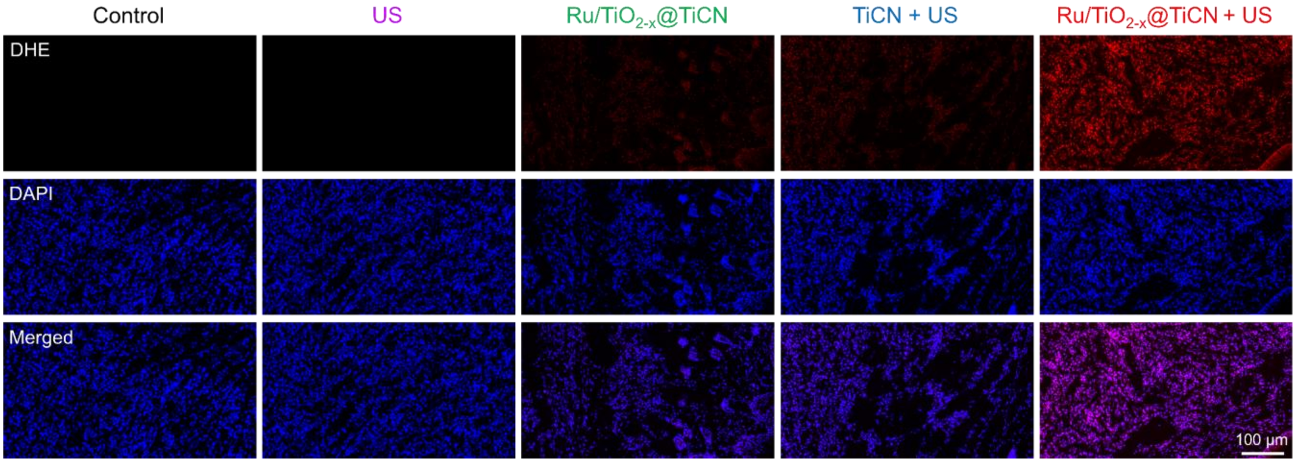


**Figure S23.** ROS staining of tumors in mice after different treatments.

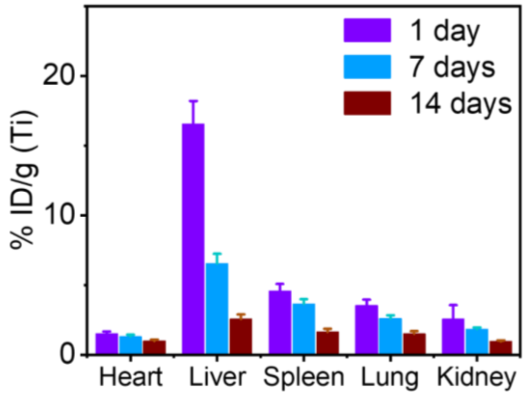


**Figure S24.** Biodistribution of Ru/$TiO_{2-x}$@TiCN post i.v. injection in mice on different days (1, 7, and 14 days) (n=5).

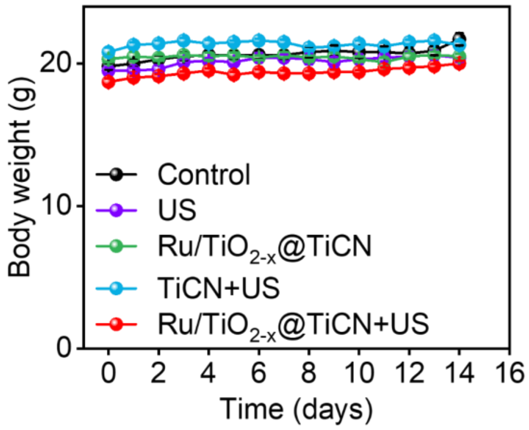


**Figure S25.** Body weight curves of the treated mice after different treatments (n=5).

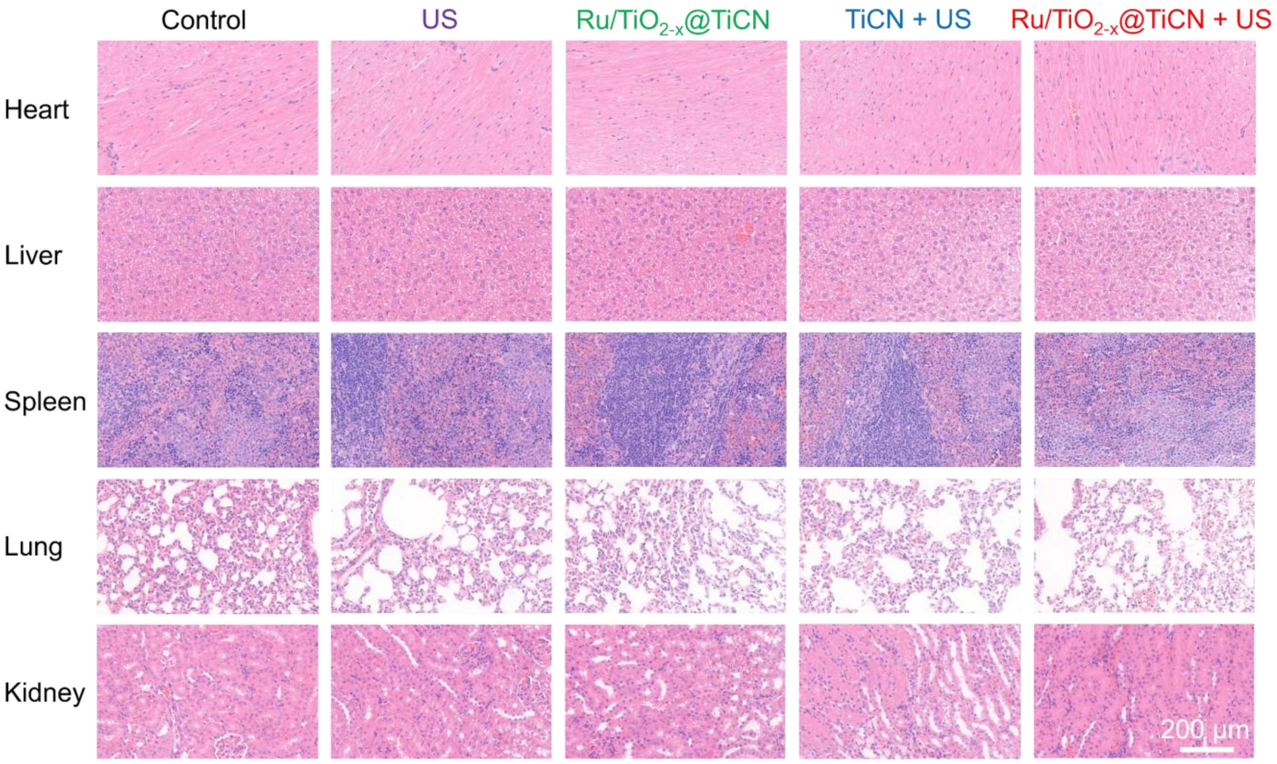


**Figure S26.** H&E-stained images obtained from the major organs (heart, liver, spleen, lung, and kidney) of mice in different treatment groups.

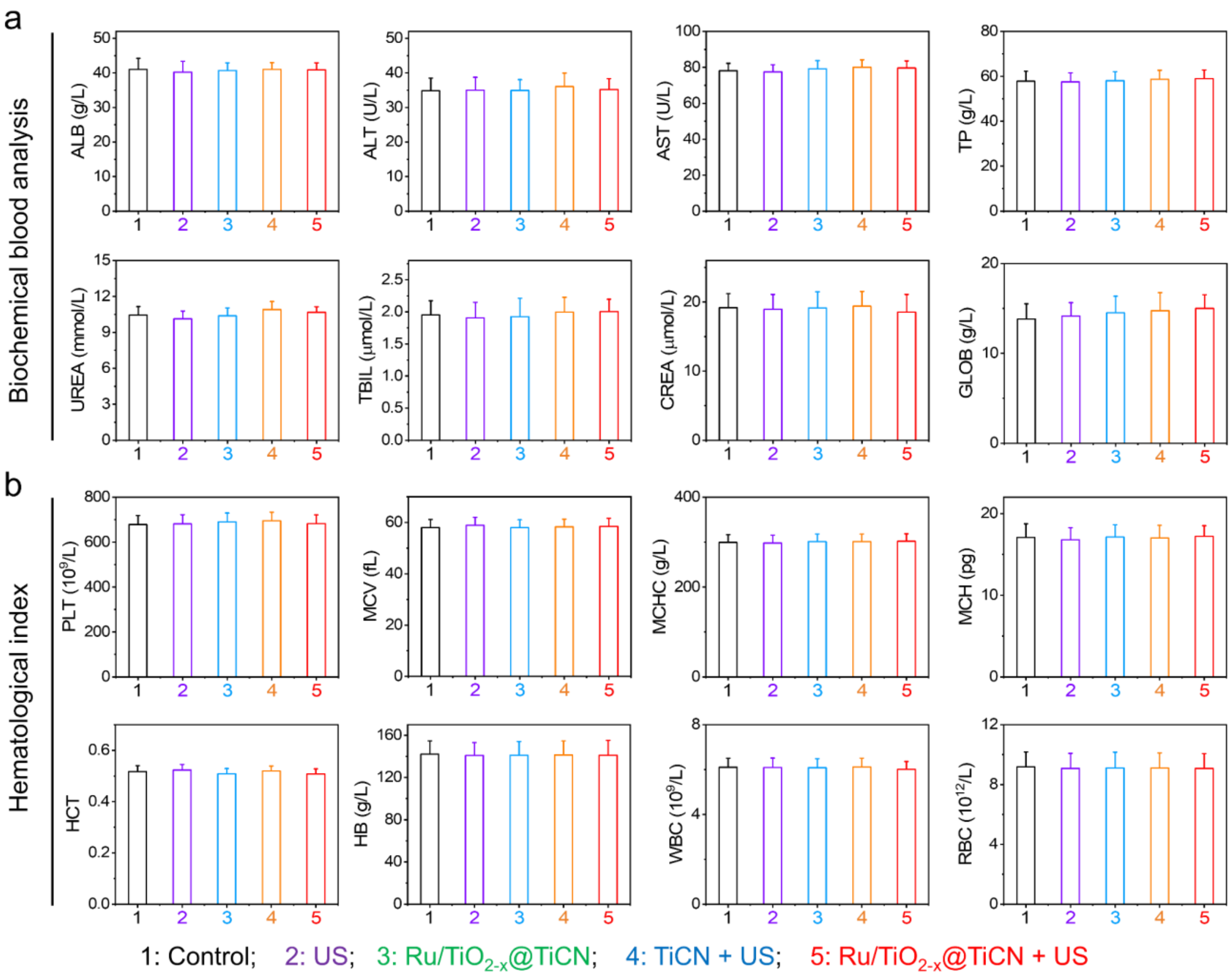


**Figure S27.** (a-b) Biochemical blood analysis (a) and hematological index (b) of the mice that were sacrificed at 18 days after different treatments (n=5).
